# Addressing non-response data for standardized post-acute functional items

**DOI:** 10.1186/s12913-023-09982-8

**Published:** 2023-09-06

**Authors:** Chih -Ying Li, Hyunkyoung Kim, Brian Downer, Mi Jung Lee, Kenneth Ottenbacher, Yong-Fang Kuo

**Affiliations:** 1 Department of Occupational Therapy, School of Health Professions, University of Texas Medical Branch, Rm 3.906, 301 University Blvd., Galveston, TX 77555-1142 USA; 2 Department of Population Health and Health Disparities, School of Public and Population Health, University of Texas Medical Branch, 301 University Blvd., Galveston, TX 77555 USA; 3 Office of Biostatistics, Department of Preventive Medicine & Public Health, University of Texas Medical Branch, 301 University Blvd., Galveston, TX 77555-1148 USA; 4 Department of Nutrition, Metabolism, and Rehabilitation Sciences, School of Health Professions, University of Texas Medical Branch, 301 University Blvd., Galveston, TX 77555 USA; 5 Sealy Center On Aging, University of Texas Medical Branch, 301 University Blvd., Galveston, TX 77555-0177 USA

**Keywords:** Functional status, Mobility, Self-care, Subacute care, Medicare payment advisory commission, Health services administration, Patient outcome assessment, Outcome and process assessment, Critical care outcomes, Health care

## Abstract

**Background:**

The post-acute patient standardized functional items (Section GG) include non-response options such as refuse, not attempt and not applicable. We examined non-response patterns and compared four methods to address non-response functional data in Section GG at nation-wide inpatient rehabilitation facilities (IRF).

**Methods:**

We characterized non-response patterns using 100% Medicare 2018 data. We applied four methods to generate imputed values for each non-response functional item of each patient: Monte Carlo Markov Chains multiple imputations (MCMC), Fully Conditional Specification multiple imputations (FCS), Pattern-mixture model (PMM) multiple imputations and the Centers for Medicare and Medicaid Services (CMS) approach. We compared changes of Spearman correlations and weighted kappa between Section GG and the site-specific functional items across impairments before and after applying four methods.

**Results:**

One hundred fifty-nine thousand six hundred ninety-one Medicare fee-for-services beneficiaries admitted to IRFs with stroke, brain dysfunction, neurologic condition, orthopedic disorders, and debility. At discharge, 3.9% (self-care) and 61.6% (mobility) of IRF patients had at least one non-response answer in Section GG. Patients tended to have non-response data due to refused at discharge than at admission. Patients with non-response data tended to have worse function, especially in mobility; also improved less functionally compared to patients without non-response data. Overall, patients coded as ‘refused’ were more functionally independent in self-care and patients coded as ‘not applicable’ were more functionally independent in transfer and mobility, compared to other non-response answers. Four methods showed similar changes in correlations and agreements between Section GG and the site-specific functional items, but variations exist across impairments between multiple imputations and the CMS approach.

**Conclusions:**

The different reasons for non-response answers are correlated with varied functional status. The high proportion of patients with non-response data for mobility items raised a concern of biased IRF quality reporting. Our findings have potential implications for improving patient care, outcomes, quality reporting, and payment across post-acute settings.

**Supplementary Information:**

The online version contains supplementary material available at 10.1186/s12913-023-09982-8.

## Brief summary

Evaluating non-response patterns of the mandatory standardized functional data and comparing the use of four handling-methods to maintain fair quality reporting and accurate determinations of needed resources and care provisions for patients across post-acute settings.

## Background

The Improving Medicare Post-Acute Care Transformation Act of 2014 (IMPACT Act) directs the Secretary of Health and Human Services to implement standardized patient assessment data elements (SPADEs) [1, 2]. The overarching goal of the IMPACT Act is to minimize cross-setting discrepancy in quality of care, data reporting and payments [1–4]; ultimately, reducing variations in Medicare expenditure while maintaining or improving clinical outcomes [5, 6]. The Centers for Medicare and Medicaid Services (CMS) includes functional data as part of the quality measures across post-acute settings: inpatient rehabilitation facilities (IRFs), skilled nursing facilities, home health agencies and long-term care hospitals [1, 2]. Section GG, part of the SPADEs, measures functional abilities and goals in self-care and mobility across post-acute settings. Currently, Section GG is directly used by the CMS to determine reimbursement for per diem rate of physical/occupational therapy and nurse services in skilled nursing facilities under the Patient-Driven Payment Model (PDPM) and IRFs. Section GG is used to calculate therapy and nursing functional scores in the Quality Reporting Programs at skilled nursing facility and IRF [7]. Technical report prepared by Acumen indicated the shift to Section GG help simplify the therapy and nursing Resource Utilization Groups (RUG) categories in PDPM due to its improved scoring accuracy. The report also found the costs associated with care were more closely aligned with Section GG than using other items such as Section G items in SAPDEs [8].

Inpatient rehabilitation programs are conventionally designed with intensive level of care to assist patients regaining function that they need for their daily lives. Healthcare practitioners at IRF typically evaluate and integrate patients’ functional data as part of care and treatment plans. The Inpatient Rehabilitation Facility Patient Assessment Instrument (IRF-PAI) [8] is used to capture patient’s daily functioning and levels of needed assistance at IRFs. Section GG items are included in the IRF-PAI after the mandatory of the IMPACT Act so all IRFs are required to report Section GG functional data beginning October 1^st^, 2016 [9–12].

Transitioning to Section GG introduces promising opportunities such as tracking functional status across settings and facilitating care coordination and cross-setting communications; eventually, improving quality of patient care and outcomes. By its design, Section GG aims to capture the full spectrum of functional performance through post-acute recovery stages. Post-acute providers are allowed to report additional non-response choices (e.g. refused) outside of the typical score range used to indicate levels of needed assistance. However, codes used in Section GG to indicate non-response options do not provide meaningful numeric labels and cannot be logically summed up (e.g. a code of ‘88’ compared to the original scale of 1–6). National Research Council [13] defined missing data as ‘…*when an outcome value that is meaningful for analysis was not collected’*. Non-response options produce values that are missing and could cause potential concerns such as inability to calculate accurate confidence intervals for the total scores, compromised statistical power, and biased parameters estimates of functional performance [14–16]. Information and evidence are currently lacking about how to adequately manage non-response data for quality reporting across post-acute settings. It raised concerns as non-response values in Section GG may lead to inaccurate determinations of needed resources and assistance, inadequate care provision and biased reimbursement determinations.

To address the concerns of using non-response options in Section GG for patient care, outcome interpretation and quality reporting, this study aims to: 1) characterize non-response patterns in Section GG, including types and frequency. We hypothesized functional levels varied among non-response options based on our clinical observations of patient functional performance; and 2) compare four methods to address non-response data and identify a relatively optimal approach to manage and report non-response data in Section GG functional items.

## Methods

### Study population

Suppl. Table 1 shows detailed cohort selection procedures. We analyzed 100% Medicare administrative claims data of patients admitted to IRFs between January 1^st^, 2018 – December 31^st^, 2018. Data were extracted on May 7^th^, 2021. The eligible cohort included Medicare fee-for-service beneficiaries ≥ 66 years old admitted to an IRF within three days of hospital discharge. Each patient had one of the following impairments: stroke, brain dysfunction, neurologic condition, orthopedic disorders, or debility. These impairments were chosen as they covered more than 90% of 2018 Medicare IRF cohort. We identified primary diagnoses and procedure codes based on the Medical Severity Diagnosis Related Groups in the Master Beneficiary Summary File and obtain functional data (both Section GG and the site-specific functional items) from the IRF-PAI file. We analyzed the first IRF stay for all patients after excluding patients who died, discharged against medical advice, or had a medical emergency during the IRF stay (Suppl. Table 1). This study followed the CMS Data Use Agreement and the University Institutional Review Board guidelines. The analyzed Medicare data comply with the Sentinel Common Data Model (SCDM) managed by the Sentinel Operations Center [17].

### Site-specific functional items: Functional Independence Measure (FIM) ®

For clarification purposes, we refer to the previous version of the IRF-PAI items as the FIM [18, 19] (site-specific) functional items versus the standardized version of the IRF-PAI items as Section GG (standardized) functional items [12, 20, 21]. However, we recognized fundamentally difference of the FIM assessment from the IRF-PAI, as the IRF-PAI comprises additional patient-level information. We analyzed six FIM self-care items (eating, grooming, toileting, bathing, dressing- upper, and dressing- lower) and five FIM mobility items (bed, chair, wheelchair (transfer), toilet (transfer), tub/shower (transfer), walk/wheelchair and stairs) in this study.

The FIM items had been collected as part of the IRF prospective payment system for decades and remained being used for payment purpose during the early stage of Section GG implementation at IRFs [22]. The FIM uses a rating scale of 1–7 to indicate the level of dependence/independence to perform functional tasks (1: total assistance, 2: maximal assistance, 3: moderate assistance, 4: minimal assistance, 5: supervision, 6: modified independence and 7: complete independence). Higher FIM scores represent more independent function. The FIM includes one non-response option of ‘not applicable’ (0) when the activity does not occur, but this code can only be used at admission [22].

### Standardized functional items: section GG

We analyzed Section GG items of IRF-PAI version 1.5 implemented at IRFs in October 2017 [20, 21], including seven self-care items (eating, oral hygiene, toileting, hygiene, shower/bathe self, upper-body dressing, lower-body dressing and put on footwear) and 17 mobility items (roll left and right, sit to lying, lying to sitting on side of bed, sit to stand, chair/bed-to-chair transfer, toilet transfer, walk 10 feet, walk 50 feet with two turns, walk 150 feet, walk 10 feet on uneven surfaces, 1 step (curb), 4 steps, 12 steps, car transfer, picking up object, wheel 50 feet with two turns, and wheel 150 feet). Section GG uses a rating scale of 1–6 to represent the level of needed assistance to perform the functional task (1: dependent, 2: substantial/maximal assistance, 3: partial/moderate assistance, 4: supervision or touching assistance, 5: setup or clean-up assistance, 6: independence). Higher Section GG scores represent more independent function. Aligning with the published CMS report [22], we used 15 of 17 mobility items (excluding two wheelchair items) to calculate total mobility scores for quality reporting purposes. Section GG self-care (*n* = 7), transfer (*n* = 6) and mobility (*n* = 15) scores were separately calculated at admission and at discharge. Detailed analyzed items can be found in the Suppl. Table 3.

### Non-response options in section GG

In version 1.5, Section GG includes three valid but non-response options to indicate answers other than the levels of needed assistance from 1 to 6. We provided each coding in the following parentheses: refuse (7), not attempt due to medical concern or safety issue (88) and not applicable (9). When no information was entered in the data field of the functional items, resulting in a dash ‘ -’ symbol in the claims, we labeled those ‘—‘ as ‘no value’. For each patient, we identified the representative non-response pattern by using the highest frequency of each reported non-response pattern (refuse, not attempt, not applicable and no value). We assigned *tie* if the patients had the same highest frequency for two non-response options.

### Handling methods for non-response functional data

To manage non-response data in functional items, we selected handling methods to (1) minimize bias, (2) maximize use of available information and (3) obtain estimates and confidence intervals of uncertainty for non-response data. Multiple imputations have been commonly used to address missing data [23]. The advantage of multiple imputations is to generate unbiased and reliable estimates of each missed value based on other available information [24]. Typically, multiple imputations involve three steps: (1) the missing value is filled out *m* times to produce *m* complete datasets using regressions based on the selected important covariates as the predictors, (2) each complete dataset is fitted with the standard procedure based on the study purpose and (3) the results separately obtained from the *m* complete datasets are combined for the estimations [25]. Data are considered as missing at random (MAR) if the likelihood of a missed value does not depend on the missing value itself but is related to other observed variables [26]. Data are considered as missing not at random (MNAR) if the probability of being missing depends on at least one unobserved variable. We applied three imputation methods, including two MAR methods (Markov Chain Monte Carlo [MCMC] and Fully Conditional Specification [FCS]) and one MNAR method (Pattern-Mixture Model [PMM]) to generate imputed values for non-response functional data. MCMC assumes that Section GG functional data are continuous while FCS assumes that Section GG functional data are categorical.

*The MCMC method* can be used for monotonic and non-monotonic missing pattern and univariate variable. MCMC generates interactable probability distributions using the Markov chain, a sequence of random variables depending on the value of the previous elements. MCMC method is based on the algorithms of simulating the joint posterior, $p(\theta |{Y}_{obs})$, for arbitrary data patterns where the underlying complete data follow the multivariate of the normal distribution [25]. With the arbitrary missing pattern and continuous Section GG data, the MCMC imputes the continuous missing values.

*The FCS method* has a different conditional distribution for each imputed variable using a Poisson model for a count variable, a logistic model or a binary variable, and a discriminant function for a categorical variable [27]. FCS imputation iterates all conditionally defined models with each iteration consisting of a single loop through all outcomes [28, 29].

*The PMM method *is based on the Bayes’ theorem to test the joint probability distribution for outcomes and missing data patterns, allowing the imputed data to be adjusted for a subset of explanatory observations. The distributions of responses in PMM are a mixture of a distribution of the observed responses and a distribution of the missing data responses. Combining with fully conditional specifications, PMM method can handle both monotone and non-monotone arbitrary missing patterns [30–33].

*The CMS method* recoded all non-response values (7, 88, 9 and dash) to 1 (the most dependent function) for Section GG items [19]. This is the published approach used to calculate quality measures with total functional scores (self-care and mobility) of Section GG data if non-response options are present.

For MCMC, FCS and PMM methods, variables in the data set used to estimate imputed values included: age, race, sex, Body Mass Index, regions, pre-hospital living setting, pre-inpatient rehabilitation facility living setting, discharge setting, the total length of IRF stay, the number of Elixhauser comorbidities, responded Section GG items, responded FIM items, and the non-response option. The imputed values were calculated by summing up values from twenty imputed datasets.

### Statistical analyses

Demographics and clinical characteristics were stratified by impairments. We compared FIM scores between patients with versus without non-response answer(s) in Section GG. We also compared FIM scores across five types of non-response patterns (refuse, not attempt, not applicable, no value and tie) to evaluate whether non-response codes represent different functional levels. We applied four methods to generate imputed values for each patient for each non-response Section GG functional item. The imputed model included covariates of age, race, sex, Body Mass Index, regions, pre-hospital living setting, pre-inpatient rehabilitation facility living setting, discharge setting, the total length of IRF stay, the number of Elixhauser comorbidities, responded Section GG items, responded FIM items, and the non-response options. Spearman coefficients and Cohen’s weighted kappa were used to examine correlations and ranking agreements (in quartiles) between Section GG and FIM before and after applying three methods. Before applying imputation methods, we excluded items with any missing prior to calculating correlation and agreement. To generate stable imputed estimates, MCMC, FCS and PMM simulated twenty datasets each and the imputed values were calculated by summing up values from twenty imputed datasets. We used MIANALYZE procedure in SAS 9.4 to generate the valid statistical inference for correlation coefficients and Cohen’s weighted kappa before and after imputations.

## Results

### Demographics

A total of 159,691 Medicare beneficiaries receiving inpatient rehabilitation services met our study criteria. The mean age was 79.1 (SD 7.5) years, the majority were non-Hispanic white (85.0%), women (56.8%), and living with family or relatives prior to hospitalization (63.9%). The most common discharge setting was home health care (57.5%), following by home self-care (23.9%) and skilled nursing facilities (17.7%). Almost half (48.9%) of the cohort with orthopedic disorders lived in the Southern states (Table 1).

**Table 1:** Demographic Characteristics by Impairment Groups (*N* = 159,691)

	**Stroke** **(*n* = 42,789)**	**Brain Dysfunction** **(*n* = 21,287)**	**Neurologic Condition** **(*n* = 22,405)**	**Orthopedic Disorders** **(*n* = 51,920)**	**Debility** **(*n* = 21,290)**
**Age at admission – CMS Calendar (years)**					
Mean (SD)	78.70 (7.44)	78.86 (7.40)	78.19 (7.19)	79.73 (7.71)	80.03 (7.63)
Median (Q1, Q3)	78 (72, 84)	78 (73, 84)	77 (72, 83)	79 (73, 86)	80 (74, 86)
**Sex, n (%)**					
Male	20,408(47.69)	11,014 (51.74)	11,054 (49.34)	16,516 (31.81)	9966 (46.81)
Female	22,381(52.31)	10,273 (48.26)	11,351 (50.66)	35,404 (68.19)	11,324 (53.19)
**Race, n (%),** missing = 1524					
Non-Hispanic white	33,946 (80.34)	17,669 (83.89)	19,216 (86.13)	45,637 (88.33)	17,964 (85.12)
Non-Hispanic Black	4498 (10.65)	1509 (7.16)	1562 (7.00)	2218 (4.29)	1673 (7.93)
Hispanic	1540 (3.64)	816 (3.87)	876 (3.93)	1766 (3.42)	719 (3.41)
Others	2268 (5.37)	1067 (5.07)	657 (2.94)	2047 (3.96)	748 (3.54)
**BMI, n (%),** missing = 247					
Underweight	1474 (3.45)	898 (4.22)	872 (3.90)	2236 (4.31)	898 (4.22)
Normal weight	14,123 (33.04)	7442 (35.01)	6503 (29.08)	16,967 (32.72)	6461 (30.38)
Overweight	14,823 (34.68)	7021 (32.48)	6909 (30.90)	16,214 (31.27)	6454 (30.35)
Obesity	12,322 (28.83)	5896 (27.74)	8076 (36.12)	16,432 (31.69)	7452 (35.04)
**Marital Status, n (%),** missing = 4702					
Never Married	4255 (10.24)	1974 (9.55)	2173 (9.93)	4320 (8.54)	2038 (9.82)
Married	20,499 (49.35)	11,080 (53.43)	11,083 (50.63)	23,049 (45.54)	9324 (44.93)
Widowed	12,890 (30.99)	5900 (28.45)	6520 (29.79)	18,794 (37.13)	7460 (35.95)
Separated	267 (0.64)	102 (0.49)	123 (0.56)	240 (0.47)	121 (0.58)
Divorced	3643 (8.77)	1682 (8.11)	1991 (9.10)	4209 (8.32)	1807 (8.71)
**Region, n (%),** missing = 883					
CT, RI, MA, ME, NH, VT	2367 (5.56)	770 (3.63)	610 (2.73)	2339 (4.54)	849 (4.00)
NY, NJ	3157 (7.41)	1213 (5.71)	782 (3.50)	3216 (6.25)	961 (4.52)
PA, WV, VA, DC, MD, DE	4880 (11.46)	2311 (10.88)	3261 (14.60)	6344 (12.32)	2473 (11.64)
KY, TN, MS, AL, GA, SC, NC, FL	9243 (21.70)	4944 (23.28)	5219 (23.37)	12,104 (23.51)	5448 (25.65)
MN, WI, MI, IL, IN, OH	7510 (17.63)	3795 (17.87)	3212 (14.38)	5382 (10.45)	3510 (16.52)
NM, OK, AR, TX, LA	6517 (15.30)	3764 (17.73)	5518 (24.71)	13,069 (25.39)	4791 (22.55)
NE, IA, KS, MO	2593 (6.09)	1257 (5.92)	1032 (4.62)	2398 (4.66)	1214 (5.71)
MT, ND, WY, SD, UT, CO	1185 (2.78)	511 (2.41)	336 (1.50)	1165 (2.26)	321 (1.51)
NV, CA, AZ	4009 (9.41)	2361 (11.12)	2276 (10.19)	5056 (9.82)	1534 (7.22)
WA, OR, ID	1128 (2.65)	309 (1.46)	89 (0.40)	409 (0.79)	142 (0.67)
**Pre-hospital Living, n (%),** missing = 2077					
Alone	12,818 (30.24)	5830 (27.82)	6681 (30.32)	18,305 (35.66)	7229 (34.57)
Family/Relative	28,009 (66.09)	14,244 (67.98)	14,486 (65.74)	31,163 (60.71)	12,848 (61.45)
Friends	558 (1.32)	210 (1.00)	251 (1.14)	446 (0.87)	200 (0.96)
Attendant	491 (1.16)	349 (1.67)	303 (1.38)	738 (1.44)	359 (1.72)
Other	507 (1.20)	321 (1.53)	313 (1.42)	682 (1.33)	273 (1.31)
**Discharge Living With, n (%),** missing = 121,525					
Alone	992 (8.28)	367 (6.86)	592 (11.76)	1385 (12.49)	710 (15.16)
Family/Relatives	9819 (81.95)	4330 (80.34)	3648 (72.48)	8182 (73.80)	3309 (70.67)
Friends	138 (1.15)	69 (1.29)	53 (1.05)	136 (1.23)	54 (1.15)
Attendant	198 (1.65)	111 (2.07)	95 (1.89)	234 (2.11)	92 (1.96)
Others^a^	835 (6.97)	505 (9.44)	645 (12.82)	1150 (10.37)	517 (11.04)
**Pre-hospital Living Setting, n (%)**					
Home^b^	42,383 (99.05)	20,954 (98.44)	22,034 (98.34)	51,334 (98.87)	20,909 (98.21)
Skilled Nursing Facility	82 (0.19)	71 (0.33)	68 (0.30)	116 (0.22)	59 (0.28)
Home Health Care	189 (0.44)	181 (0.85)	177 (0.79)	261 (0.50)	223 (1.05)
Others^a^	135 (0.32)	81 (0.38)	126 (0.56)	209 (0.40)	99 (0.47)
**Admitted to Rehab From, n (%)**					
Home^b^	349 (0.82)	213 (1.00)	343 (1.53)	587 (1.13)	253 (1.19)
Short-term Hospital	42,113 (98.42)	20,907 (98.21)	21,751 (97.08)	50,807 (97.86)	20,854 (97.95)
Skilled Nursing Facility	85 (0.20)	62 (0.29)	90 (0.40)	170 (0.33)	76 (0.36)
Others^a^	242 (0.57)	105 (0.49)	221 (0.99)	356 (0.69)	107 (0.50)
**Discharge Setting, n (%)**					
Home^b^	11,982 (28)	5352 (25.14)	5033 (22.46)	11,087 (21.35)	4682 (21.99)
Skilled Nursing Facility	10,491 (24.52)	3520 (16.54)	2682 (11.92)	9233 (17.78)	2290 (10.76)
Home Health Care	19,921 (46.56)	12,098 (56.83)	14,434 (64.42)	31,280 (60.25)	14,068 (66.08)
Others^a^	395 (0.92)	317 (1.49)	256 (1.14)	320 (0.62)	250 (1.17)
**Number of Elixhauser comorbidity**					
Mean (SD)	3.36 (1.40)	3.11 (1.43)	3.22 (1.49)	2.71 (1.40)	3.29 (1.43)
Median (Q1, Q3)	3 (2, 4)	3 (2, 4)	3 (2, 4)	3 (2, 4)	3 (2, 4)

^a^Others were included Short-term General Hospital, Hospice (home and institutional facility), Another Inpatient Rehabilitation Facility, Long-Term Care Hospital (LTCH)

^b^Home was included the private home/apt., board/care, assisted living, group home, transitional living

^c^All demographic characteristics of patients were significantly different by impairment groups

### Non-response patterns in section GG

Patients with different diagnoses showed non-response answers at admissions and discharge. The same patient tended to have more complete responses on the FIM than on the Section GG (Suppl. Table 2). When examining Section GG alone, more patients tended to have non-response answers at admission than at discharge (e.g., missing any item in self-care: 16.4% at admission and 3.9% at discharge). At discharge, the percent of patients with any non-response options in Section GG self-care, transfer and mobility was 3.9%, 5.6% and 61.6%, respectively. An extremely high percent of patients showed at least one non-response answer in mobility items (95.4%, 61.5% and 96.0% for admission, discharge, and change, respectively) (Suppl. Table 2). The most common non-response item at discharge was the mobility abilities ‘to go up and down 12 steps with or without a rail’ (38.2%, without considering two wheelchair items) (Suppl. Table 3). Suppl. Figure 1 demonstrated the percent of each non-response option in each Section GG item at admission and discharge.

Table 2 shows FIM scores for patients with and without any non-response values and for those with any non-response values stratified by five non-response types. For all participants, the most common non-response answer was ‘*not attempt’* (except for self-care at admission where ‘refused’ was the most common) and the least common answer was ‘*no value’* (if not considering ‘tie’) (Table 2). More patients had non-response data due to ‘*refused’* at discharge than at admission. The same trend was found across impairments (Suppl. Table 4, using stroke as an example as other impairments showed similar patterns).

**Table 2:** Functional Independence Measure Scores between Patients with and without Non-Response Section GG Options and by Non-Response Type (*N* = 159,691)

	**All,** Mean (SD), Median (Q1, Q3)		**With Non-Response,** Mean (SD), Median (Q1, Q3)				
	**Without Non-Response**	**With Non-Response**	**Tie **^**a**^	**No-Tie**			
				**No Value**	**Refused**	**Not Attempted**	**Not Applicable**
***Admission***							
**GG SC**							
N (%)	133,518 (83.6)	26,173 (16.4)	929 (3.6)	29 (0.1)	8997 (34.4)	13,241 (50.6)	2977 (11.4)
FIM SC	19.2 (5.8), 20 (15, 24)	17.2 (6.1), 17 (13, 22)	15.9 (5.9), 16 (12, 20)	18.4(6.0), 19 (13, 22)	18.1 (5.8), 18 (14, 22)	16.6(6.2), 17 (12, 21)	17.8 (6.0), 18 (14, 22)
**GG Trans**							
N (%)	134,170 (84.0)	25,521 (16.0)	582 (2.3)	21 (0.1)	3958 (15.5)	19,019 (74.5)	1941 (7.6)
FIM Trans	7.6 (3.1), 8 (5, 10)	5.9 (3.0), 5 (3, 8)	5.4(2.7), 4 (3, 7)	5.9 (3.1), 5 (3, 8)	6.3(3.0), 6 (3, 9)	5.7 (2.9), 5 (3, 8)	7.0(3.2), 7 (4, 9)
**GG MO**							
N (%)	7321 (4.6)	152,370 (95.4)	4394 (2.9)	21,666 (14.2)	1994 (1.3)	120,907 (79.4)	3409 (2.2)
FIM MO	15.7 (5.0), 17 (12, 20)	10.27 (4.1), 10 (7, 13)	10.5 (4.7), 10 (6, 14)	6.6 (2.4), 6 (5, 8)	11.7(4.2), 11 (8, 14)	10.8 (4.0), 11 (8, 14)	12.7 (4.7), 13 (9, 16)
***Discharge***							
**GG SC**							
N (%)	153,424 (96.1)	6267 (3.9)	100 (1.6)	34 (0.6)	2682 (42.8)	2588 (41.3)	863 (13.8)
FIM SC	31.7 (7.1), 33 (28, 37)	25.2 (9.0), 26 (19, 32)	21.2(8.9), 21.5 (15, 28)	26.5(10.2), 26 (18, 35)	26.6(8.3), 27 (21, 33)	23.4 (9.5), 24 (16, 31)	26.6 (8.4), 28 (21, 33)
**GG Trans**							
N (%)	150,538 (94.3)	9153 (5.7)	125 (1.4)	27 (0.3)	1480 (16.2)	6355 (69.4)	1166 (12.7)
FIM Trans	14.4 (3.8), 15 (12, 18)	11.2 (5.3), 12 (6, 16)	7.2 (4.4), 6 (3, 11)	11.6(5.2), 12 (7, 17)	10.8(5.0), 11 (7, 15)	11.1 (5.4), 12 (6, 16)	12.3 (4.7), 13 (9, 16)
**GG MO**							
N (%)	61,495 (38.5)	98,196 (61.5)	4868 (5.0)	8440 (8.6)	4753 (4.8)	70,724 (72.0)	9411 (9.6)
FIM MO	26.6 (4.3), 28 (24, 30)	19.7 (6.6), 20 (15, 25)	20.8(5.9), 22 (18, 25)	12.2(6.4), 11 (6, 18)	21.6 (6.1), 22 (18, 26)	20.1 (6.2), 21 (16, 25)	22.5 (5.7), 23 (19, 27)
***Change (Discharge- Admission)***							
**GG SC**							
N (%)	130,297 (81.6)	29,394 (18.4)	N/A^b^	N/A^b^	N/A^b^	N/A^b^	N/A^b^
FIM SC	12.7 (6.0), 13 (9, 17)	11.9 (6.6), 12 (8, 16)	N/A^b^	N/A^b^	N/A^b^	N/A^b^	N/A^b^
**GG Trans**							
N (%)	130,093 (81.5)	29,598 (18.5)	N/A^b^	N/A^b^	N/A^b^	N/A^b^	N/A^b^
FIM Trans	7.0 (3.4), 7 (5, 9)	6.4 (4.1), 7 (3, 9)	N/A^b^	N/A^b^	N/A^b^	N/A^b^	N/A^b^
**GG MO **^**C**^							
N (%)	6383 (4.0)	153,308 (96.0)	N/A^b^	N/A^b^	N/A^b^	N/A^b^	N/A^b^
FIM MO	11.2(4.6), 11 (8, 14)	11.9 (5.6), 12 (8, 16)	N/A^b^	N/A^b^	N/A^b^	N/A^b^	N/A^b^

*Abbreviation*: *SC* Self-Care, *MO* Mobility, *Trans* Transfer, *FIM* Functional Independence Measure

^a^The primary type of non-response option was determined based on the highest frequency of non-response type for Section GG. If patient had same frequency of two more types, this case was considering as ‘Tie’ category

^b^Change score of GG was calculated as discharge score minuses admission scores (e.g. GGSC Change = Discharge GGSC – Amission GGSC). Thus, we did not have information on patients with non-response data

^c^The GG mobility score at admission and discharge is created using 15 GG items, excluding two wheelchair items

Patients with at least one non-response answer in Section GG had lower FIM scores (more functionally dependent), especially in mobility, and improved less functionally compared to patients without non-response answer. Across five non-response answers, patients coded as ‘*refused’* in self-care overall had the highest FIM scores (more functionally independent). Patients coded as ‘*not applicable’* overall had the highest FIM scores (more functionally independent) in transfer and mobility compared to other non-response answers (Table 2).

### Comparisons of four handling methods

Overall, correlations between Section GG and FIM increased after addressing the missing data using any of the four methods, but it is unclear if the change is large enough to be determined as clinically significant (Table 3). Changes in correlations varied across impairments after applying four methods. Changes in correlations were more consistent among imputation methods themselves (MCMC, FCS and PMM) compared to CMS method but overall all four methods showed similar results, with three exceptions (all compared to the original values before imputation): (1) correlations increased for admission mobility in stroke but decreased in brain injury, orthopedic disorders, neurological condition and debility after applying MCMC, FCS and PMM, but not CMS approach; (2) correlations decreased for admission mobility in orthopedic disorders (original r = 0.80) after applying MCMC, FCS and PMM (r = 0.68) but slightly decreased after applying the CMS approach (r = 0.77); (3) correlations slightly decreased for admission mobility in neurological disorders (original r = 0.72) after applying MCMC, FCS and PMM (r = 0.69) but slightly increased after applying CMS approach (r = 0.77) (Table 3).

**Table 3:**
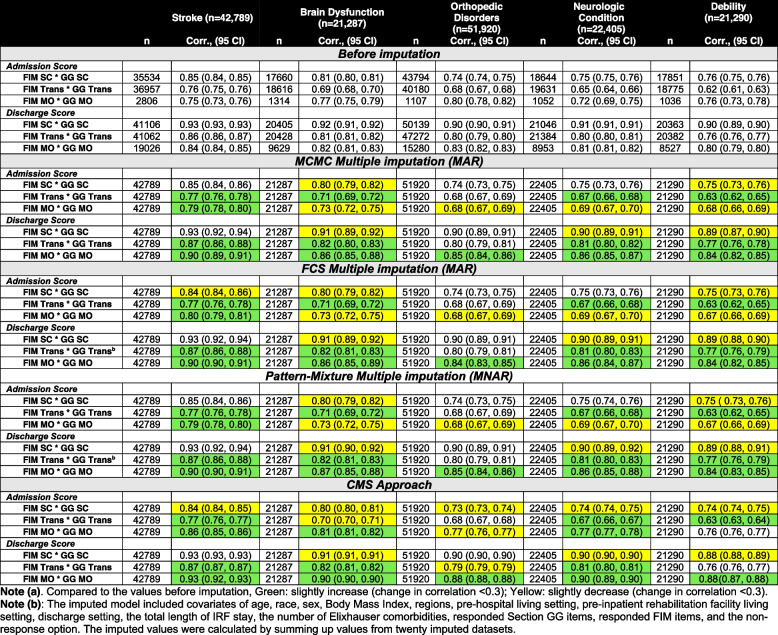
Correlation by Impairment before and after Applying Three Non-Response Data Handling-Methods (*N* = 159,691)

*Abbreviation*: *MAR* Missing at random, *MNAR* Missing not at random, *SC* Self-Care, *MO* Mobility, *Trans* Transfer, *FIM* Functional Independence Measure, *MCMC* Monte Carlo Markov Chains multiple imputations, *FCS* Fully Conditional Specification multiple imputations, *CMS* Centers for Medicare and Medicaid Services

^a^Compared to the values before imputation, Green: slightly increase (change in correlation < 0.3); Yellow: slightly decrease (change in correlation < 0.3)

^b^The imputed model included covariates of age, race, sex, Body Mass Index, regions, pre-hospital living setting, pre-inpatient rehabilitation facility living setting, discharge setting, the total length of IRF stay, the number of Elixhauser comorbidities, responded Section GG items, responded FIM items, and the non-response option. The imputed values were calculated by summing up values from twenty imputed datasets

Similar to the correlation findings, agreements overall improved between Section GG and FIM after addressing the missing data using any of four methods (Table 4). We did not observe any significant drops but found significant improvement in agreement after addressing the missing data for discharge mobility in stroke, orthopedic disorders and neurological disorders (improved ≥ 0.3 unit of weighted kappa). Similar as the correlation findings, changes of agreement varied across impairments after applying four methods. However, overall changes in agreements were similar when applying four methods. Only one difference was observed between imputation methods and CMS approach: self-care and transfer at discharge in brain dysfunction, with agreements slightly decreased after applying MCMC, FCS and PMM methods (self-care: original weighted kappa = 0.74; MCMC/FCS/PMM = 0.72) but slightly improved after applying the CMS approach (CMS = 0.75) (Table 4). Finally, we found Imputation methods showed similar results regardless of using MAR or MNAR approaches.

**Table 4:**
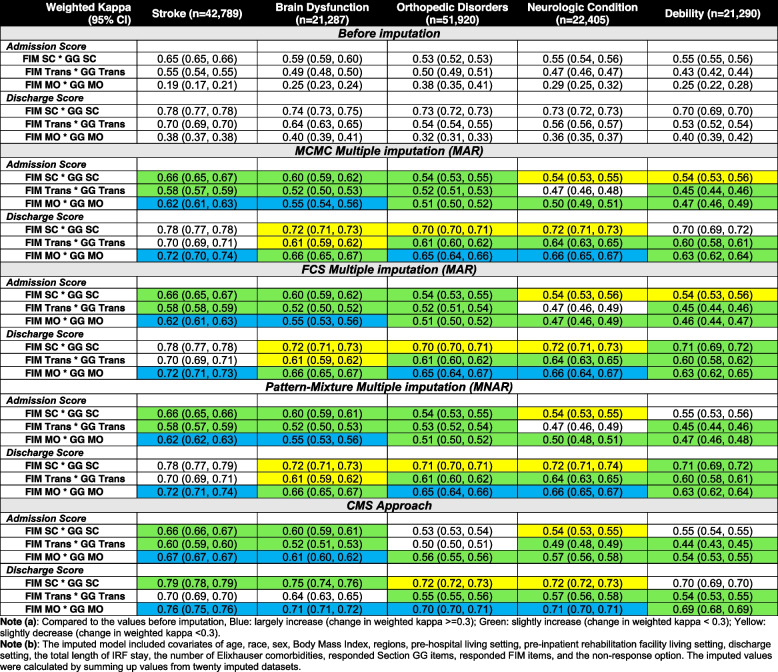
Agreement by Impairment before and after Applying Three Non-Response Data Handling-Methods (*N* = 159,691)

*Abbreviation*: *MAR* Missing at random, *MNAR* Missing not at random, *SC* Self-Care, *MO* Mobility, *Trans* Transfer, *FIM* Functional Independence Measure, *MCMC* Monte Carlo Markov Chains multiple imputations, *FCS* Fully Conditional Specification multiple imputations, *CMS* Centers for Medicare and Medicaid Services

^a^Compared to the values before imputation, Blue: largely increase (change in weighted kappa >  = 0.3); Green: slightly increase (change in weighted kappa < 0.3); Yellow: slightly decrease (change in weighted kappa < 0.3)

^b^The imputed model included covariates of age, race, sex, Body Mass Index, regions, pre-hospital living setting, pre-inpatient rehabilitation facility living setting, discharge setting, the total length of IRF stay, the number of Elixhauser comorbidities, responded Section GG items, responded FIM items, and the non-response option. The imputed values were calculated by summing up values from twenty imputed datasets

## Discussion

This study examines non-response answers in Section GG, with a goal to better characterize and manage non-response Section GG functional data. There is limited information in current literature that directly examined non-response Section GG data. However, it was noted in 2019 Medicare Post-Acute Care Commission (MedPAC) report that a high percent of non-response Section GG data was discovered and removed from the MedPAC analysis. The report found home health agencies had the most incomplete data (30%) as the items of eating and toileting hygiene were often missed from four analyzed functional items. Skilled nursing facilities, on the other hand, had the most completed patient assessment data (only 1% missing of those four functional items) [34]. Consistently, we found a very high percent of patients with at least one non-response code for Section GG mobility performance, indicating the need and importance to develop a systematic and feasible way to manage non-response data especially for Section GG mobility items at IRFs. We also found functional levels varied among non-response answers in Section GG. Patients with any non-response code in Section GG were more functionally dependent particularly in mobility compared to patients who did not miss any. The most common non-response answer was ‘*not attempt*’ and the least common answer was ‘*no value*’, implying clinicians kept patient safe while reporting the functional data as much as they could. The direction of associations between Section GG and FIM is similar across all methods, but there are differences in magnitude of the associations among the methods. We found similar changes after applying four methods to manage non-response data, but three imputation methods (MCMC, FCS, and PMM) generated more consistent results among themselves compared to the CMS approach.

Different non-response answers showed varied levels of assistance that patients need when performing functional tasks. For instance, patients coded as ‘*refused*’ tended to be more functionally independent in self-care compared to other non-response answers. CMS currently recode all non-response answers in Section GG to the same least independent functional level “1” for quality measure calculations. This approach does not consider varied levels of needed assistance across non-response options. It is uncertain if this ‘one-size-fits-all’ approach is adequate as clinical observations and our findings supported that functional needs and care varied for patients who refused (e.g. due to emotional distress or dissatisfied), who did not attempt to perform a functional task (e.g. due to medical safety concerns), or who simply could not perform the task (e.g. did not have the capacity). Recoding all non-response answers to the same functional level (1, least functional) although is quick and easy but may lead to inaccurate score calculations and further biased distributions of needed resources and qualified reimbursement. As a result, patients may not receive needed support and limit their opportunity to recover fully and ultimately may prevent the patients from developing life skills and live independently. The CMS approach may thus widen disparities in health care and service provisions.

Patients care the most after discharge from acute care is to regain their daily function. Studies also found being able to perform self-care and mobility functional tasks such as eating, dressing, grooming and bed mobility significantly predicts post-acute 30-day and 90-day hospital readmission risk [35–37]. It is imperative for all post-acute stakeholders to be onboard and properly report the levels of needed assistance so patients can independently perform daily functional tasks. Given the high prevalence of non-response answers reported in Section GG functional items, practitioners and policy makers urgently need a feasible method to accurately adjust for and report the needed assistance when non-response data were reported. Surprisingly, we found CMS approach seemed to improve correlation and agreement with a relatively equivalent effect as either the MCMC, FCS, or PMM multiple imputation methods. We suggested CMS approach might be used when managing non-response Section GG data given its similar results as imputation methods. However, the issue of different functional levels embedded in different non-response options still need to be recognized and addressed when using the CMS approach.

This study extends the examination of the Section GG data from our previous work [12] and further evaluated non-response functional data with four handling methods. We expect our work can facilitate ongoing conversations among across post-acute stakeholders to identify an optimal way to adequately evaluate and reflect the needed assistance and quality reporting for patients with non-response answers in Section GG items. Our findings should encourage CMS to continue developing a payment system that can addresses potential health disparities and expand our understanding of how to fairly manage non-response Section GG data. This study sets up baseline evidence to further determine the impact of non-response functional data on quality reporting and payment across post-acute settings.

### Limitation

The used CMS approach may have an advantage of having better correlation and agreement results with FIM given the similar approach was also applied in the site-specific FIM functional items (CMS recoded 0 ‘not applicable’ to 1 ‘total dependent’ in FIM to calculate discharge total FIM scores). Thus, our finding may overestimate and favor the strength of the CMS approach. We also recognize that IRF-PAI has been frequently updated, and the most current version is the IRF-PAI v.4.0 implemented at IRFs on October 1^st^, 2020 [38]. The Section GG items in IRF-PAI v.1.5 analyzed in this study were implemented in October 2017 when the mobility items were not instructed to be skipped. However, the analyzed Section GG items in our study were essentially the same as the Section GG items in the most updated versions of the IRF-PAI. Our findings in detecting high percent of non-response answers in Section GG mobility items support the use of skipped patterns that is currently implemented in the updated versions of IRF-PAI. We also recognized that methods used to impute missing data have been advanced over the past decade. More advanced imputation methods such as Item Response Theory imputation [39] and a nested multiple imputation [40] may provide a new insight to interpret missing data differently and account for potential errors of using ordinal rating scales, but these methods have limited usability and generalizability. We selected the commonly applied imputation methods in this study with a consideration that not all innovative imputation methods are currently widely recognized or understood, to allow a broader audience to replicate our findings using the same multiple imputation methods.

## Conclusions

Our findings provide practical information to assist interpreting and addressing non-response answers in Section GG for practice, research and policy making. Section GG data are intended to be used for quality reporting of care across post-acute settings. Non-response answers in Section GG are inevitable and should be adequately managed so the levels of assistance that patients need for their independent living can be properly addressed. Current CMS approach may be an effective way to manage non-response Section GG data given its similar findings as sophisticated multiple imputation methods. We support the need to develop a more granular and systematic approach to manage different functional levels among non-response answers, to fulfill the goal of better assisting different needs of daily functioning for patients. Future studies are needed to identify whether non-response answers in Section GG impact on the aspects of patient care, outcomes, quality reporting, and payment across post-acute settings.

### Supplementary Information

**Additional file 1:** **Suppl. Table 1.** Cohort Selection Flow Chart. **Suppl. Table 2.** Percent of Non-Response Data in Section GG and FIM by Domain and Evaluation Time (*N*=159,691). **Suppl. Table 3.** Percent of Non-Response Data in Each Item of Section GG and FIM at Admission and Discharge (*N*=159,691). **Suppl. Table 4.** Site-Specific Functional Scores for Patients with and without Non-Response Data and by Non-Response Type (Stroke) (*N*=42,789). **Suppl. Figure 1.** Percent of Each Non-Response Option in Each Section GG Item at Admission and Discharge.

## Data Availability

The Research Data Assistance Center (ResDAC) maintains a high level of data security to protect patient privacy for Medicare enrollees. The Data Use Agreement with ResDAC is required to access CMS data. Medicare data files supporting the conclusions of this article are thus not publicly available. Interested readers can access the analyzed data through ResDAC at: https://resdac.org Detailed data purchasing, and Data Use Agreement information can be available in the ResDAC website.

## Abbreviations

CMS: Centers for Medicare and Medicaid Services
FCS: Fully Conditional Specification
FIM®: Functional Independence Measure
IMPACT Act: Improving Medicare Post-Acute Care Transformation Act of 2014
IRF: Inpatient Rehabilitation Facility
IRF-PPS: Inpatient Rehabilitation Facility Prospective Payment System
IRF-PAI: Inpatient Rehabilitation Facility Patient Assessment Instrument
MedPAC: Medicare Payment Advisory Commission
MAR: Missing at Random
MCMC: Markov Chain Monte Carlo
MNAR: Missing Not at Random
PAC: Post-acute Care
PMM: Pattern-Mixture Model
SPADEs: Standardized patient assessment data elements
