# Supplementary material for: Addressing non-response data for standardized post-acute functional items
Source: BMC Health Serv Res. 2023 Sep 6;23:955. doi: 10.1186/s12913-023-09982-8 (PMC10481609; doi:10.1186/s12913-023-09982-8)
Supplement: Supplementary file 1 — Additional file 1: Suppl. Table 1. Cohort Selection Flow Chart. Suppl. Table 2. Percent of Non-Response Data in Section GG and FIM by Domain and Evaluation Time (N=159,691). Suppl. Table 3. Percent of Non-Response Data in Each Item of Section GG and FIM at Admission and Discharge (N=159,691). Suppl. Table 4. Site-Specific Functional Scores for Patients with and without Non-Response Data and by Non-Response Type (Stroke) (N=42,789). Suppl. Figure 1. Percent of Each Non-Response Option in Each Section GG Item at Admission and Discharge. [file 12913_2023_9982_MOESM1_ESM.docx]

**Supplementary Files**

**Suppl. Table 1. Cohort Selection Flow Chart**

| Step |  | N | % |
| --- | --- | --- | --- |
| 1 | Select the first IRF in 2018 | 427902 |  |
| 2 | Select IRF patients within 3 day of acute case stay | 308,312 | 78.3% |
| 3 | Exclude the length of stay is less than 3 days in IRF. | 300,935 | 97.6% |
| 4 | Select IRF patients older than 66 on date of IRF admission | 247,411 | 82.2% |
| 5 | Select five impairment conditions (stroke, brain dysfunction, neurologic condition, orthopedic disorders and debility) | 225,457 | 91.1% |
| 6 | Select survived IRF patients | 225,107 | 99.8% |
| 7 | Exclude IRF patients discharged against medical advice | 224,494 | 99.7% |
| 8 | Exclude IRF patients who had medical emergency* | 204,347 | 91.0% |
| 9 | Exclude IRF patients who were discharged directly to another IRF | 204,166 | 99.9% |
| 10 | Select patients who were continuously enrolled in Part A for the 12months before IRF stay admission date | 159,691 | 78.2% |
| *Patients who had medical emergency defined as patients discharged to the following destinations: short-term general hospital, long-term care hospital, inpatient psychiatric facility, and critical access hospital. | | | |

**Suppl. Table 2. Percent of Non-Response Data in Section GG and FIM by Domain and Evaluation Time (N=159,691).**

| Functional Independence Measure | | | |
| --- | --- | --- | --- |
|  | Admission | Discharge | Change |
| Self-Care | 5.9% | 0.0% | 5.9% |
| Transfer | 33.1% | 0.0% | 33.1% |
| Mobility | 60.8% | 0.0% | 60.8% |
| Section GG | | | |
|  | Admission | Discharge | Change |
| Self-Care | 16.4% | 3.9% | 18.4% |
| Transfer | 16.0% | 5.7% | 18.5% |
| Mobility | 95.4% | 61.5% | 96.0% |

**Suppl. Table 3. Percent of Non-Response Data in Each Item of Section GG and FIM at Admission and Discharge (N=159,691).**

|  |  | **Section GG** | |  | **Functional Independence Measure** | |
| --- | --- | --- | --- | --- | --- | --- |
| **Domain** |  | Admission  (% of non-response) | Discharge (% of non-response) |  | Admission (% of non-response) | Discharge (% of non-response) |
| **Self-Care** | Eating | 1.3% | 0.6% | Eating | 0.1% | 0% |
|  | Oral Hygiene | 1.3% | 0.3% | Grooming | 0.4% | 0% |
|  | Toileting Hygiene | 2.5% | 0.4% | Toileting | 1.3% | 0% |
|  | Shower/bathe self | 4.0% | 0.5% | Bathing | 3.4% | 0% |
|  | Upper-body dressing | 0.7% | 0.2% | Dressing- upper | 1.3% | 0% |
|  | Lower-body dressing | 0.6% | 0.2% | Dressing- lower | 0.7% | 0% |
|  | Put on footwear | 1.2% | 0.5% |  |  |  |
| **Mobility** | Roll left and right * | 4.8% | 2.3% | Bed, chair, wheelchair (transfer)* | 0.2% | 0% |
|  | Sit to lying * | 1.2% | 0.4% | Toilet (transfer) * | 3.1% | 0% |
|  | Lying to sitting on side of bed * | 0.9% | 0.4% | Tub/shower (transfer) * | 32.4% | 0% |
|  | Sit to stand * | 1.8% | 0.8% |  |  |  |
|  | Chair/bed-to-chair transfer * | 0.9% | 0.3% |  |  |  |
|  | Toilet transfer * | 6.1% | 1.4% |  |  |  |
|  | Walk 10 feet | 20.6% | 6.0% | Walk/wheelchair | 5.0% | 0% |
|  | Walk 50 feet with two turns | 46.8% | 11.9% |  |  |  |
|  | Walk 150 feet | 71.0% | 22.2% |  |  |  |
|  | Walk 10 feet on uneven surfaces | 72.6% | 26.6% |  |  |  |
|  | 1 step (curb) | 55.5% | 17.2% | Stairs | 48.7% | 0% |
|  | 4 steps | 62.2% | 21.5% |  |  |  |
|  | 12 steps | 83.1% | 38.2% |  |  |  |
|  | Car transfer | 58.5% | 13.1% |  |  |  |
|  | Picking up object | 69.3% | 31.9% |  |  |  |
|  | Wheel 50 feet with two turns | 44.3% | 44.1% |  |  |  |
|  | Wheel 150 feet | 60.4% | 49.2% |  |  |  |

*: Transfer items in FIM and Section GG were selected for this study; &: Two wheelchair items were excluded in Section GG mobility total scores.

**Note**: Official full IRF-PAI questionnaire (version 1.5) can be directly accessed at: <https://www.cms.gov/Medicare/Quality-Initiatives-Patient-Assessment-Instruments/IRF-Quality-Reporting/Downloads/Proposed-IRF-PAI-Version-15-Effective-October-1-2017.pdf>

**Suppl. Table 4. Site-Specific Functional Scores for Patients with and without Non-Response Data and by Non-Response Type (Stroke) (N=42,789).**

|  | **All,** Mean (SD), Median (Q1, Q3) | | **With missing,** Mean (SD), Median (Q1, Q3) | | | | |
| --- | --- | --- | --- | --- | --- | --- | --- |
|  | **Without missing** | **Missing** | **Tie^a^** | **No Tie^b^** | | | |
|  |  |  |  | **No Value** | **Refused** | **Not Attempted** | **Not Applicable** |
| ***Admission Score*** |  |  |  |  |  |  |  |
| **GG SC** |  |  |  |  |  |  |  |
| N (%) | 35534 (83.0) | 7255 (17.0) | 262 (3.6) | 18 (0.3) | 2028 (28.0) | 4069 (56.1) | 878 (12.1) |
| FIM SC | 18.6 (6.6), 19 (14, 24) | 15.8 (6.8), 15 (10, 21) | 14 (6.2), 13 (9, 19) | 17.4 (5.6), 19 (12, 22) | 17.6 (6.4), 18 (13, 23) | 14.8 (6.8), 14 (9, 20) | 16.3 (6.8), 16 (10, 22) |
| **GG Trans** |  |  |  |  |  |  |  |
| N (%) | 36957 (86.4) | 5832 (13.6) | 103 (1.8) | 8 (0.1) | 882 (15.1) | 4351 (74.6) | 488 (8.4) |
| FIM Trans | 7.7 (3.4), 8 (5, 11) | 5.3 (3.1), 4 (3, 7) | 4.7 (2.7), 3 (3, 6) | 4.8 (2.3), 4 (3, 6) | 6.4 (3.2), 6 (3, 9) | 5.0 (2.9), 3 (3, 6) | 6.9 (3.4), 6 (3.5, 9.5) |
| **GG MO^C^** |  |  |  |  |  |  |  |
| N (%) | 2806 (6.6) | 39983 (93.4) | 1094 (2.7) | 6454 (16.1) | 361 (0.9) | 31164 (77.9) | 910 (2.3) |
| FIM MO | 16.7 (4.6), 18 (14, 20) | 10.4 (4.5), 10 (6, 14) | 10.7 (5.3), 10 (5, 15) | 6.0 (1.9), 5 (5, 6) | 13.2 (4.6), 13 (9, 17) | 11.2 (4.3), 11 (8, 14) | 13.6 (5.0), 14 (10, 18) |
| ***Discharge Score*** |  |  |  |  |  |  |  |
| **GG SC** |  |  |  |  |  |  |  |
| N (%) | 41106 (96.1) | 1683 (3.9) | 28 (1.7) | 11 (0.7) | 592 (35.2) | 797 (47.4) | 255 (15.2) |
| FIM SC | 29.8 (7.9), 31 (25, 36) | 21.9 (9.5), 22 (14, 30) | 18.4 (8.0), 20 (11.5, 24) | 20.6 (9.4), 20 (17, 26) | 25.4 (8.6), 26 (19, 32) | 18.8 (9.2), 18 (10, 26) | 23.7 (9.1), 24 (18, 31) |
| **GG Trans** |  |  |  |  |  |  |  |
| N (%) | 41062 (96.0) | 1727 (4.0) | 23 (1.3) | 6 (0.4) | 277 (16.0) | 1177 (68.2) | 244 (14.1) |
| FIM Trans | 13.6 (4.3), 15 (12, 17) | 8.0 (5.2), 6 (3, 12) | 5.1 (3.7), 3 (3, 7) | 9.7 (6.0), 8.5 (5, 15) | 9.5 (4.9), 9 (5, 13) | 7.1 (5.1), 4 (3, 12) | 10.7 (5.2), 11 (5, 15) |
| **GG MO** |  |  |  |  |  |  |  |
| N (%) | 19026 (44.5) | 23763 (55.5) | 975 (4.1) | 2579 (10.9) | 916 (3.9) | 17084 (71.9) | 2209 (9.3) |
| FIM MO | 26.0 (4.5), 26 (24, 29) | 17.7 (7.2), 18 (12, 23) | 19.0 (6.5), 20 (16, 24) | 8.8 (4.8), 7 (5, 10) | 21.4 (6.1), 22 (18, 25.5) | 18.2 (6.6), 18 (14, 23) | 22.0 (6.1), 23 (18, 26) |
| ***Change Score*** |  |  |  |  |  |  |  |
| **GG SC** |  |  |  |  |  |  |  |
| N (%) | 34722 (81.2) | 8067 (18.9) | N/A^b^ | N/A^b^ | N/A^b^ | N/A^b^ | N/A^b^ |
| FIM SC | 11.5 (6.0), 11 (7, 15) | 10.6 (6.6), 10 (6, 15) | N/A^b^ | N/A^b^ | N/A^b^ | N/A^b^ | N/A^b^ |
| **GG Trans** |  |  |  |  |  |  |  |
| N (%) | 36191 (84.6) | 6598 (15.4) | N/A^b^ | N/A^b^ | N/A^b^ | N/A^b^ | N/A^b^ |
| FIM Trans | 6.2 (3.4), 6 (4, 9) | 4.8 (4.0), 5 (1, 8) | N/A^b^ | N/A^b^ | N/A^b^ | N/A^b^ | N/A^b^ |
| **GG MO** |  |  |  |  |  |  |  |
| N (%) | 2581 (6.0) | 40208 (94.0) | N/A^b^ | N/A^b^ | N/A^b^ | N/A^b^ | N/A^b^ |
| FIM MO | 10.5 (4.4), 11 (8, 13) | 10.5 (5.7), 11 (7, 14) | N/A^b^ | N/A^b^ | N/A^b^ | N/A^b^ | N/A^b^ |

**Abbreviation:** SC: Self-Care; MO: Mobility; Trans: Transfer. FIM: Functional Independence Measure. **Note**: a. The majority types of non-response would be considered as the type of non-response category for Section GG. If patient had same frequency of two more types, this case would be considering as ‘Tie’ category. b. Change score of GG was created using admission and discharge score: GGSC Change = Discharge GGSC – Admission GGSC. Thus, we did not have information on patients with non-response data. c. The GG mobility score at admission and discharge is created using 15 GG items, excluding two wheelchair items.

**Suppl. Figure 1. Percent of Each Non-Response Option in Each Section GG Item at Admission and Discharge.**

| **Section GG Self-Care** |
| --- |
| 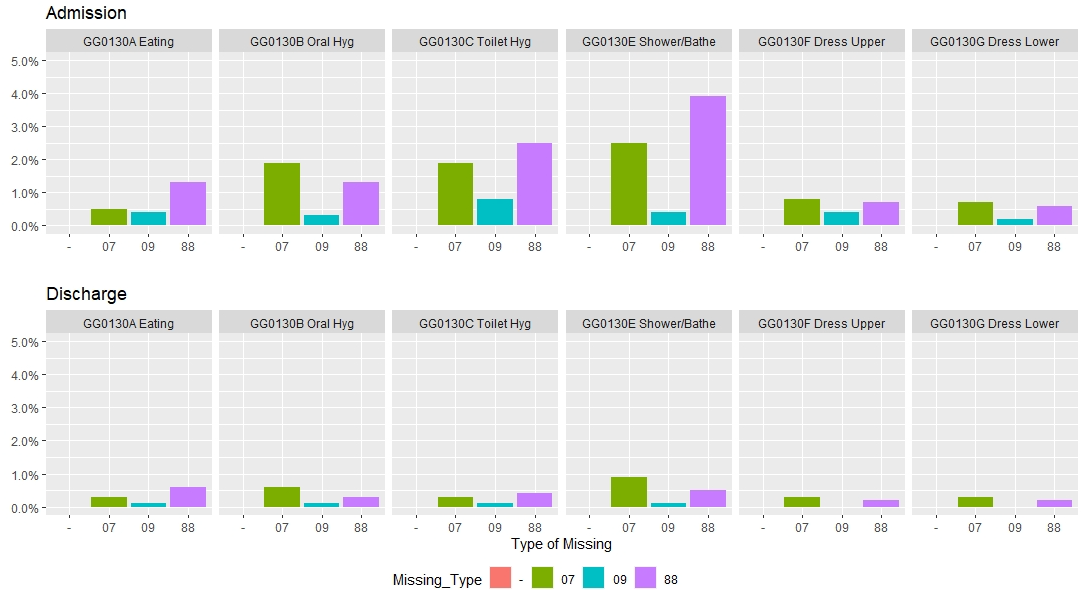  **Note:** Red: no value (-); Green: refuse (07), Blue: not applicable (09), Purple: not attempt due to medical concern or safety issue (88). |
| **Section GG Mobility** |
| 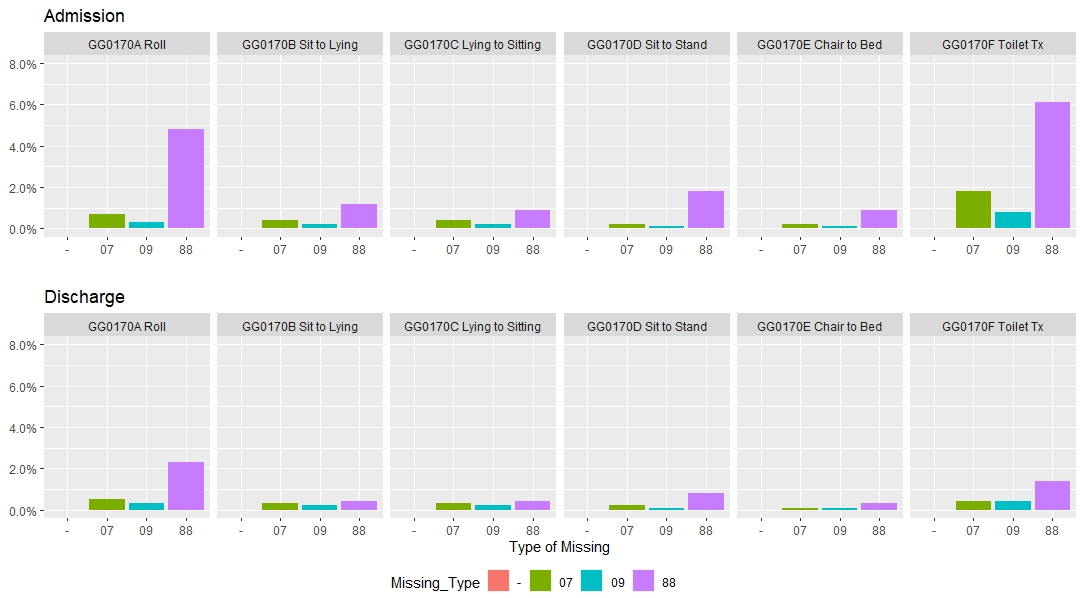  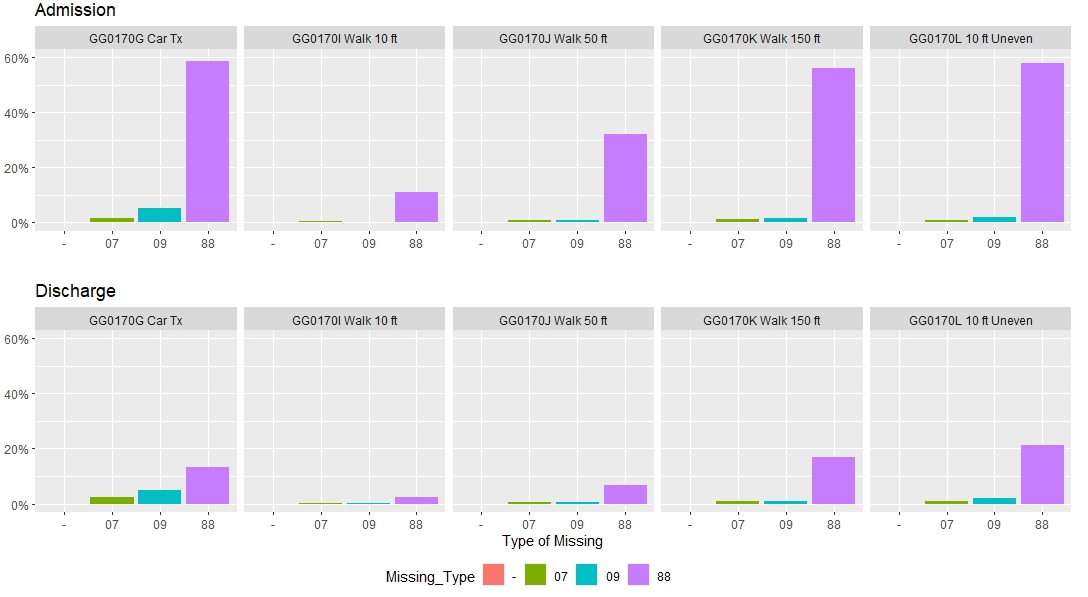  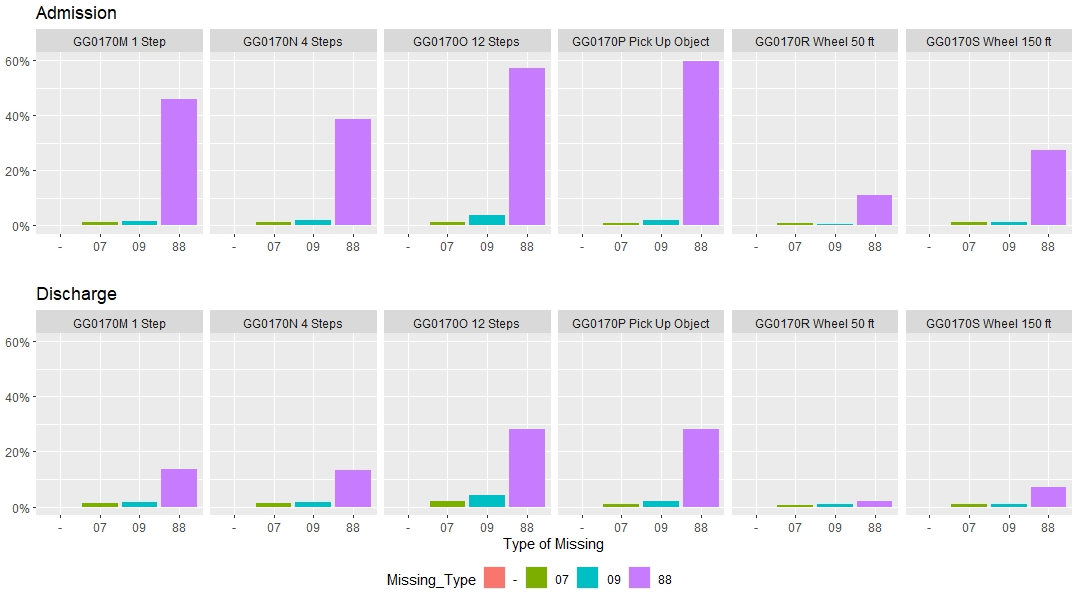  **Note:** Red: no value (-); Green: refuse (07), Blue: not applicable (09), Purple: not attempt due to medical concern or safety issue (88). |
